# Addiction consult service involvement in PrEP and PEP delivery for patients who inject drugs admitted to an urban essential hospital

**DOI:** 10.1186/s13722-024-00502-5

**Published:** 2024-11-04

**Authors:** Hallie Rozansky, Paul J. Christine, Morgan Younkin, Jason M. Fox, Zoe M. Weinstein, Sebastian Suarez, Jessica Stewart, Natalija Farrell, Jessica L. Taylor

**Affiliations:** 1 Section of General Internal Medicine, Boston University Chobanian and Avedisian School of Medicine and Boston Medical Center, 801 Massachusetts Avenue, Second Floor, Boston, MA 02118 USA; 2 Grayken Center for Addiction, Boston Medical Center, Boston, MA USA; 3 Division of General Internal Medicine, University of Colorado School of Medicine, Aurora, CO USA; 4 Boston Healthcare for the Homeless Program, Boston, MA USA; 5 Division of Hospital Medicine, University of Miami Miller School of Medicine, Miami, FL USA; 6 Center for Infectious Diseases, Boston Medical Center, Boston, MA USA; 7 Department of Quality and Patient Safety, Boston Medical Center, Boston, MA USA; 8 Department of Emergency Medicine, Boston University Chobanian and Avedisian School of Medicine, Boston, MA USA

**Keywords:** Pre-exposure prophylaxis, HIV prevention, Opioid use disorder, People who inject drugs

## Abstract

**Background:**

Addiction medicine providers have a key role in HIV prevention amidst rising HIV incidence in persons who inject drugs (PWID). Pre-exposure prophylaxis (PrEP) and post-exposure prophylaxis (PEP) are vastly underutilized in this population. Inpatient hospitalization represents a potential touchpoint for initiation of HIV prophylaxis, though little research explores the role of addiction providers. Here we describe rates of PrEP/PEP delivery to hospitalized PWID seen by an Addiction Consult Service (ACS) at an urban, essential hospital.

**Methods:**

We performed a cross-sectional study of hospitalized patients who were seen by the ACS from January 1, 2020 to December 31, 2022 and had plausible injection drug use. We calculated the proportion of patients who received a new prescription for PrEP/PEP at discharge. We used descriptive statistics to characterize demographics, substance use, reason for admission, and indications for PrEP/PEP. Secondarily, we calculated the monthly proportion of all patients discharged from the hospital with PrEP/PEP who were seen by the ACS compared to those not seen by the ACS.

**Results:**

The average monthly proportion of ACS consults with plausible injection drug use who received PrEP/PEP was 6.4%. This increased from 4.2% in 2020 to 7.5% in 2022. Those seen by the ACS who received PrEP/PEP had high rates of opioid use disorder (97.5%), stimulant use disorder (77.8%), and homelessness (58.1%); over half were admitted for an injection-related infection. The indications for PrEP/PEP were injection drug use only (70.6%), followed by combined injection and sexual risk (20.2%); 71.9% of prescriptions were for PrEP and 28.1% for PEP. Overall, the ACS was involved in 83.9% of hospital-wide discharges with PrEP/PEP prescriptions (n = 242).

**Conclusions:**

PWID who were seen by the ACS received PrEP/PEP prescriptions at rates exceeding national averages. The ACS was also involved with the care of the majority of admitted patients who received PrEP/PEP at discharge. While PrEP/PEP use for PWID remains low, the inpatient ACS represents a key resource to improve uptake by leveraging the reachable moment of an inpatient hospitalization.

## Background

HIV outbreaks among persons who inject drugs (PWID) and other people who use drugs have been a morbid consequence of the opioid and polysubstance use crises [1], occurring in rural [2] and urban regions, and in areas with and without robust harm reduction infrastructure [3]. PWID are at high risk of HIV acquisition, accounting for about 10% of HIV diagnoses in the United States [4]. Up to 1 in 42 PWID will contract HIV in their lifetime [5]; in comparison, the lifetime risk of acquiring HIV through any means is approximately 1 in 76 for men and 1 in 309 for women [6]. HIV pre-exposure prophylaxis (PrEP) prevents HIV transmission among PWID [7], and real-world observational studies also support the effectiveness of HIV post-exposure prophylaxis (PEP) in PWID [8, 9]. However, PrEP and PEP remain vastly underutilized. A recent study demonstrated that among persons with commercial insurance and likely injection drug use, only 0.15% received PrEP based on outpatient pharmacy fill data [10]; other data have shown rates of uptake between 0 and 5% in this population [1, 11].

Inpatient hospitalization is a critical touchpoint for people who use drugs [12, 13], who may experience barriers to accessing preventive services in outpatient settings [14]. Many PWID are hospitalized for infectious complications of drug use [15–17], opening the door to conversations about infection prevention and risk reduction, including PrEP [18]. PrEP and PEP are safe and easy to prescribe. However, there is wide institutional variability in whether HIV prevention counseling and management is done inpatient and by whom. A recent retrospective cohort study demonstrated that of 300 HIV-negative patients hospitalized with infectious complications of IDU, 55% of whom were seen by an ACS, only 2 had documented discussions about PrEP [13]. Studies in outpatient and inpatient settings have demonstrated varying levels of comfort prescribing PrEP for PWID among both general internists and infectious disease providers [19–21].

The availability of inpatient Addiction Consult Services (ACS), staffed by addiction medicine and/or psychiatry clinicians providing subspecialty consultation on hospitalized patients with addiction-related medical conditions, has grown over the years [22, 23]. These services are uniquely positioned to support evidence-based HIV prevention services for hospitalized PWID, including PrEP and PEP, given their existing focus on harm reduction. To our knowledge, only one other study has described the use of PrEP and PEP in hospitalized PWID cared for by an inpatient ACS [13].

To this end, our study aimed to evaluate PrEP and PEP delivery to hospitalized patients cared for by an ACS at an urban, essential hospital in Boston, MA, a city with rising incidence of HIV among PWID [24], located in one of the federal *Ending the HIV Epidemic* priority counties [25]. We aimed to assess the monthly rate of hospitalized PWID seen by our ACS who were discharged with a PrEP or PEP prescription and describe trends in demographics and substance use characteristics among these patients. Secondary objectives were to quantify the monthly proportion of all hospitalized patients who received PrEP and PEP that were seen by our ACS, in order to understand the involvement of our ACS with HIV prevention efforts hospital-wide.

## Methods

### Site

Boston Medical Center is an urban, essential hospital located in one of the federal *Ending the HIV Epidemic* priority counties and is also located at the epicenter of the substance use epidemic in Boston [26]. At our institution, there were between 44 to 60 new HIV diagnoses annually during the study period; PWID accounted for an average of 26% of these new diagnoses (unpublished data).

The ACS at Boston Medical Center is a multidisciplinary inpatient consult team, which provides recommendations to primary inpatient teams regarding initiation of medications to treat substance use disorders and also provides counseling and support, harm reduction education, and assistance transitioning to community-based addiction treatment programs after hospital discharge [22]. In 2019, increased HIV transmission was noted among PWID in Boston [24], and the MA DPH and Boston Public Health Commission began to alert providers and recommend consideration of PEP and PrEP for this population shortly after. In response, the ACS began more systematically discussing and offering PrEP and PEP to patients, modifying its note template in the electronic medical record (EMR) to include discussion of pharmacologic HIV prevention as a standard harm reduction measure. While the ACS does not directly write orders for patients, team members provide recommendations, including regarding initiation of PrEP or PEP, to primary teams.

### Study population and analytic sample

For our primary analyses evaluating rates of PrEP and PEP discharge prescriptions to patients seen by our ACS, we used EMR reports to identify all inpatient ACS consults that could plausibly involve injection drugs (and hence qualify for PrEP or PEP for that indication) between January 1, 2020 and December 21, 2022. We defined plausible injection drug use if the consult order referenced opioid or stimulant use (as injecting is the primary route of opioid and stimulant use in our patient population), or specifically referenced injecting drugs. From this list of consult episodes, we focused on those who were discharged with new prescriptions containing tenofovir disoproxil fumarate (TDF) for in-depth chart review, as TDF (in combination with emtricitabine) is the standard PrEP for PWID at our institution. With regard to other medications used for PrEP, tenofovir alafenamide (TAF) has less robust data in PWID and is thus rarely used for this indication at our institution, and cabotegravir is not on our inpatient formulary, so we did not evaluate patients discharged on these medications. We excluded patients on TDF for treatment of HIV or Hepatitis B. Readmissions were counted as separate episodes in the sample.

For secondary analyses designed to understand the total proportion of patients prescribed PrEP/PEP hospital-wide who were seen by the ACS, we used discharge pharmacy records to identify all inpatients ≥ 18 years old who were discharged with TDF during the study period, again excluding those on TDF for treatment of HIV and Hepatitis B.

### Variables of interest

For patients seen by ACS and discharged on PrEP or PEP, we collected the following data: sociodemographics (age, gender, race/ethnicity, housing status), drug use history (presence of opioid, stimulant, benzodiazepine, alcohol, or other substance use disorders (SUD), history of overdose), reason for admission (injection-related infection vs. other), length of stay (days), whether a patient left the hospital as a patient-directed discharge (historically known as “against medical advice”; yes/no), indication for PrEP or PEP (injection drug use, sexual risk, or both), and whether PrEP or PEP was a new prescription or a continuation of prior therapy. Chart reviews were performed by two of the authors (HR, PC) and any ambiguities around diagnoses or variable coding were discussed until consensus was achieved, with the assistance of a third author (JT) when necessary.

### Statistical analysis

To assess rates of prescribing among eligible patients seen by the ACS, we calculated the monthly proportion of all inpatient ACS consults that involved plausible injection drug use and received PrEP or PEP prescriptions at discharge. We then calculated descriptive statistics and measures of dispersion for those patients who received PrEP or PEP to better characterize this population, using means with standard deviations or medians with interquartile ranges for continuous variables and proportions for categorical variables.

In secondary analyses designed to understand the contribution of the ACS to PrEP and PEP prescribing hospital-wide, we calculated the monthly proportion of all patients discharged on PrEP or PEP that were seen by the ACS during their hospital stay compared to inpatients who were not seen by ACS.

Analyses were conducted using R version 4.2.2. This study was deemed exempt by the Boston University Medical Campus Institutional Review Board [H-41111].

## Results

Among the patients seen by ACS who received PrEP or PEP (n = 162 unique patients), the median age was 38 years and patients were more commonly male (54.2%) and non-Hispanic White (65.5%) (Table 1). Over half of hospitalization episodes involved patients who were unhoused (58.1%). Most patients in this sample had opioid use disorder (OUD) (97.5%), followed by stimulant use disorders (77.8% overall; 67% cocaine, 35% methamphetamine). Concomitant benzodiazepine and alcohol use disorders were also common (33.5% and 27.1%, respectively). For hospitalizations involving individuals with OUD, 92.9% were discharged on medications for opioid use disorder (MOUD), with methadone being most common (79.3%) followed by buprenorphine (20.7%).

**Table 1:** **Characteristics of inpatient addiction consult episodes where patients were prescribed PrEP/PEP for HIV**

Characteristic	Outcome
Overall episodes, n	203
Unique individuals, n (%)	162 (79.8)
Age, median (IQR)	38 (32, 42)
Gender, n (%)	
Female	93 (45.8)
Race/ethnicity, n (%)	
White, non-hispanic	133 (65.5)
Black, non-hispanic	36 (17.7)
Hispanic, all races	33 (16.3)
Other/not reported	1 (0.5)
Primary language, n (%)	
English	200 (98.5)
Spanish	2 (1.0)
Other	1 (0.5)
Currently unhoused, n (%)	118 (58.1)
Substance use disorders, n (%)	
Opioid use disorder	198 (97.5)
Stimulant use disorder	158 (77.8)
Cocaine	136 (67.0)
Amphetamines	71 (35.0)
Benzodiazepine use disorder	68 (33.5)
Alcohol use disorder	55 (27.1)
Other drug use disorder	22 (10.8)
History of drug overdose, n (%)	171 (84.2)
Prophylaxis type, n (%)	
PrEP	146 (71.9)
PEP	57 (28.1)
Indication for prophylaxis, n (%)	
Injection drug use only	144 (70.9)
Sexual risk only	17 (8.4)
Injection drug use and sexual risk	41 (20.2)
Not documented	1 (0.5)
Prophylaxis new or continuation, n (%)	
New prescription	173 (85.2)
Continuation of prior prescription	30 (14.8)
Admitted for injection related infection, n (%)	111 (54.7)
Left hospital as patient-directed discharge, n (%)	26 (12.8)
Length of hospital stay (days), median (IQR)	4 (1. 9)
Patient with OUD discharged on MOUD, n (%)*	184 (92.9)
Methadone	146 (79.3)
Buprenorphine	38 (20.7)
Naltrexone	0 (0)

^*^Denominator is total number of episodes with OUD, n = 198

**Fig. 1:**
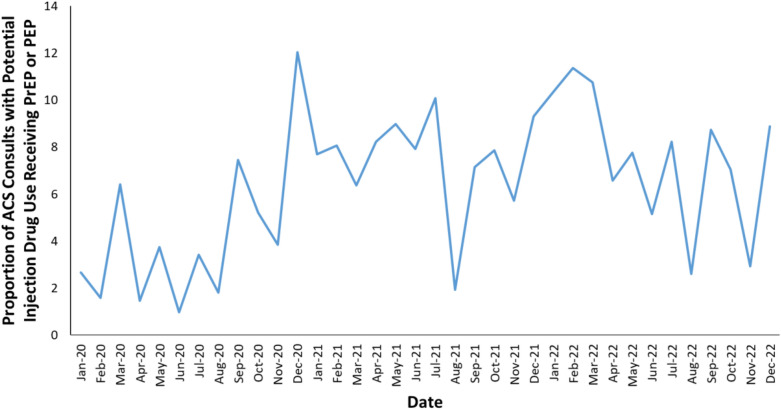
**Temporal trends in addiction consults with plausible injection drug use that received PrEP/PEP at discharge.** Consults with potential injection drug use included any consult for opioid use disorder, stimulant use disorder, or consults that specifically mentioned injection drug use

Our primary analysis demonstrated that the average monthly proportion of patients with plausible injection drug use who were seen by the ACS and received PrEP or PEP was 6.4% (range 1.0% to 12.0%; Fig. 1). This proportion increased over time from an average of 4.2% of consults per month in 2020 to 7.5% of consults per month in 2022.

Most prescriptions were for PrEP (71.9%) rather than PEP (28.1%), and the most common indication was injection drug use (70.9%), followed by combined injection drug use and sexual risk (20.2%) (Table 1). Most PrEP or PEP prescriptions were new starts (85.2%), rather than a continuation of a prior prescription, and a small but notable proportion of episodes received PrEP or PEP despite leaving as a patient-directed discharge (12.8%). Additionally, among this sample, over half of admissions were for injection-related infections (54.7%).

**Fig. 2:**
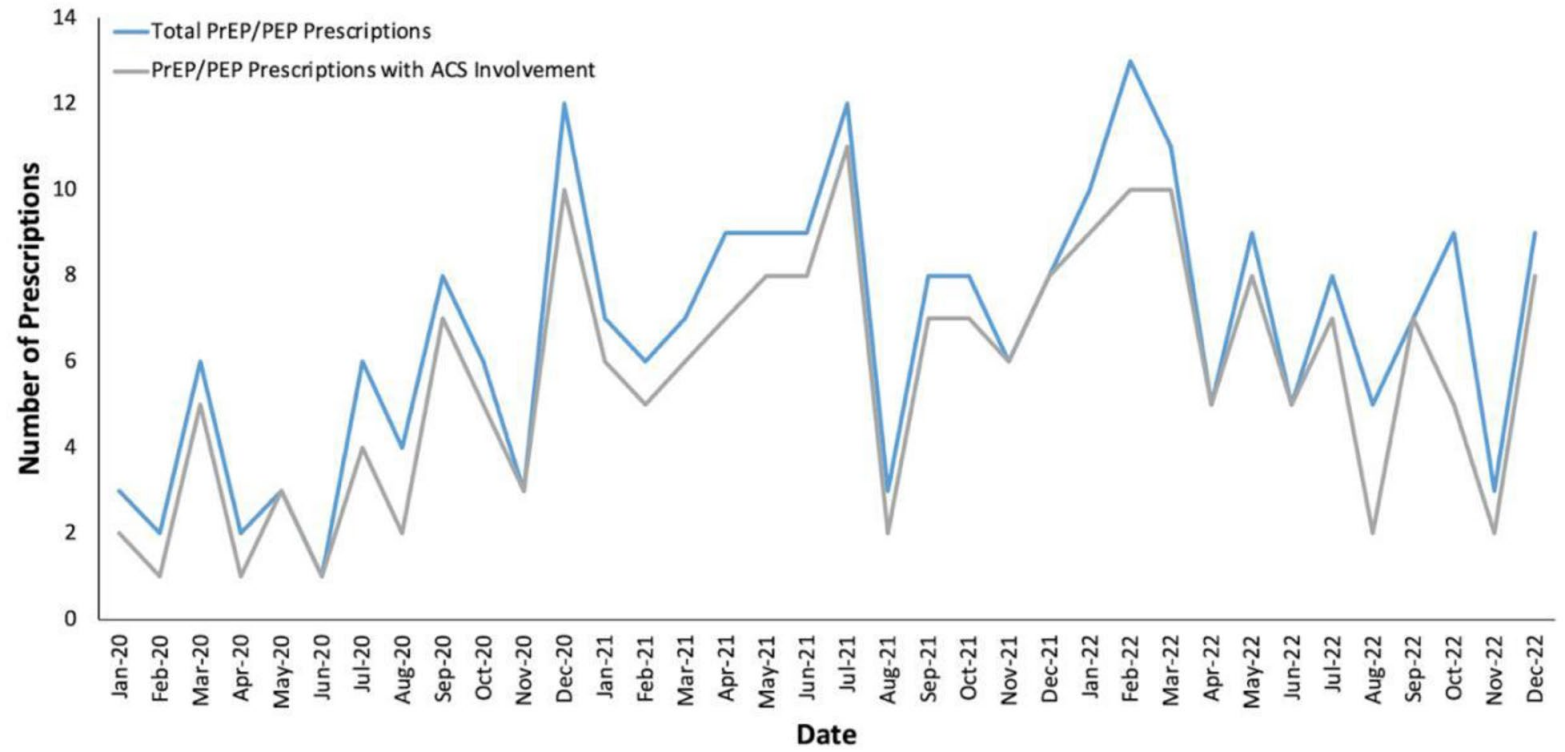
**Temporal trends in PrEP/PEP prescriptions, stratified by ACS involvement**

Our secondary analysis demonstrated that there were 242 total hospitalizations where patients were prescribed PrEP or PEP at discharge, and the ACS was involved in 203 of these (83.9%, 162 unique patients). Monthly proportions of ACS involvement in PrEP/PEP prescriptions ranged from 40 to 100% (Fig. 2), and the number of monthly hospitalization episodes with a discharge prescription for PrEP or PEP increased over time from an average of 4.7 (SD 3.1) in 2020 to an average of 7.8 (SD 2.9) in 2022 (Fig. 2; t-test for difference in means comparing 2020 and 2022, p = 0.02). There was considerable month-to-month variation in the number of prescriptions, ranging from 1 to 13.

## Discussion

In this urban, essential hospital located in a city with high HIV burden among PWID, an average of 6.4% of patients seen by the ACS with plausible injection drug use were prescribed PrEP or PEP at time of discharge. This proportion increased over time, from 4.2% in 2020 to 7.5% in 2022. At lowest, it was on par with the higher end of national averages, and at highest, exceeded published rates [1, 10, 11]. Secondarily, the ACS was involved in the care of the vast majority (83.9%) of admitted patients who received PrEP or PEP at discharge.

To our knowledge, this is the second study describing PrEP and PEP prescribing to patients seen by an ACS [13], and demonstrates promise for leveraging the inpatient ACS to increase uptake of pharmacologic HIV prophylaxis in an at-risk population with high rates of overdose, homelessness, and polysubstance use. This complements the ways in which the ACS supports other evidence-based forms of HIV prevention, including harm reduction counseling [27], provision of new injection equipment [28], and initiation of gold-standard MOUD, such as methadone and buprenorphine [29]. A high proportion (92.9%) of patients in our study who were seen by the ACS and received PEP or PrEP also received MOUD at time of hospital discharge. Additional data on ACS harm reduction services are needed to fully characterize contributions to HIV prevention.

Secondarily, we demonstrated that the ACS was involved in most hospitalizations that included a PrEP or PEP discharge prescription. While the ACS does not write orders for PrEP and PEP directly, our EMR note template for the ACS explicitly prompts providers to ask patients about eligibility for PrEP and PEP and provide recommendations accordingly. Furthermore, our addiction medicine fellows, who work on the ACS, receive formal teaching on PrEP and PEP for PWID. This is important in light of data demonstrating that many medical providers hesitate to provide PrEP or PEP to PWID due to lack of comfort or knowledge [19–21]; anecdotally, there is also ambiguity about which specialty should “own” PrEP or PEP, particularly in the inpatient setting. Though we cannot demonstrate causality between ACS involvement and PrEP/PEP prescribing, our data lends support to the value of the ACS in stepping up to fill a potential void, and the inclusion of PEP and PrEP within addiction specialists’ scope of practice. As such, other institutions aiming to increase PrEP and PEP prescribing could consider similar, systematic interventions to support the role of an ACS in this space (e.g., developing note templates, implementing formal education on PrEP and PEP in PWID).

Inpatient hospitalization has been previously described as a “reachable moment” to provide care to patients who may have difficulty accessing traditional outpatient medical settings, including as a means to link patients to MOUD after nonfatal overdoses and other complications of OUD [12, 30]. Incorporating pharmacologic HIV prevention into the scope of practice of an ACS capitalizes on this opportunity, particularly when patients are already hospitalized with infectious complications of SUD, as in our sample, where over 50% were admitted for an injection-related infection. While PrEP has been traditionally considered an outpatient medication, for high-risk hospitalized patients with SUD, there are benefits to PrEP initiation prior to the post-acute care transition [18].

This study has several limitations. First, the goal of our study was primarily descriptive and we therefore do not include a comparison time period when the ACS did not exist or a comparison group of PWID without ACS involvement. While a control group would have been ideal, each of these would have been represented very different sets of patients from which appropriate comparisons could not be drawn. The ACS at our institution has been in existence since 2015, and substance use patterns and HIV prevalence among PWID have changed substantially since that time. For instance, since 2015, fentanyl has become ubiquitous in the opioid drug supply and concurrent stimulant use has become common [31, 32], both of which drive an increased number of injection events per day. Additionally, a 2015–2018 HIV outbreak among PWID in Lawrence and Lowell, MA, cities close to Boston, prompted a Massachusetts Department of Public Health and CDC EpiAid investigation and raised local awareness about the need for increased PEP and PrEP delivery to PWID [33]. Thus a “pre-ACS” control group would have represented a very different population than that included in the present study. In terms of comparing patients seen versus not seen by the ACS, the patients seen by the ACS typically represent those with more unstable SUD and higher-risk IDU. Even with propensity matching, it would be difficult to build a control cohort with a similar degree of medical complexity, severity of addiction, and burden of psychosocial barriers. In future studies seeking to estimate causal effects of ACS involvement on PrEP and PEP utilization, the use of an appropriate control group should be considered.

Second, in our study, demonstration of causality is further limited since the ACS provides recommendations to primary teams but does not directly prescribe medications. However, documentation from ACS notes supports recommendations and indications for PrEP and PEP for the majority of these patients. Third, the patients who received PrEP or PEP and were seen by the ACS were primarily White and English-speaking. We lacked race/ethnicity data on the larger patient population seen by the ACS overall and were thus unable to compare patients who received PrEP or PEP to all patients seen by the ACS. These disparities potentially reflect well-documented inequities in SUD care delivery [34]. Additionally, the racial and language breakdown of our study population is consistent with the population of PWID accessing care at our institution, indicating a broader opportunity to improve SUD care delivery—including PrEP and PEP—to diverse populations who inject drugs.

Fourth, patients were categorized as using a substance only if their chart had documentation to support a use disorder by Diagnostic and Statistical Manual V criteria, which may have underestimated prevalence of SUD. Fifth, when identifying the indication for PrEP and PEP, sexual risk may have been less thoroughly evaluated and documented by clinicians; anecdotally, we observed more consistent evaluation of injection-related risk. This theory is supported by a recent study of PrEP and PEP eligibility in an outpatient SUD bridge clinic setting, showing that while 85.7% of patients were assessed for injection-related risk, only 23% were assessed for sexual risk [35]. Sixth, we abstracted data for patients prescribed TDF/FTC, the form of PrEP most commonly used for PWID at our institution, but did not evaluate patients prescribed TAF/FTC or cabotegravir off-label, and thus may have underestimated PrEP delivery. Seventh, we excluded patients with Hepatitis B, since initiation of PrEP in these patients requires a more complex risk/benefit discussion and at our institution, is typically deferred to a specialty team; we chose to focus on PrEP initiations clearly within the scope of the ACS. Finally, while we document considerable month-to-month variation in the receipt of PrEP or PEP for likely eligible patients, we lacked data on clinician and patient factors that may contribute to such variability, as well as patient refusals, which may be significant [36]. Understanding these factors is important in potentially increasing the receipt of PrEP and PEP during hospitalization.

## Conclusion

At a large, urban, essential hospital in a city with a rising incidence of HIV among PWID, patients seen by the ACS received PrEP and PEP at rates comparable to or exceeding the national average, with most receiving it for the indication of injection drug use and many also receiving it for concurrent injection and sexual risk. The ACS was involved in the care of the majority of admitted patients who received PrEP and PEP at time of discharge. ACS are thus well-positioned to advocate for and optimize HIV prevention, including encouraging PrEP and PEP utilization, and may be able to assist in leveraging the reachable moment of an inpatient hospitalization to improve rates of pharmacologic HIV prevention uptake.

## Data Availability

N/A.

## Abbreviations

ACS: Addiction consult service(s)
EMR: Electronic medical record
MOUD: Medications for opioid use disorder
PWID: Persons who inject drugs
PrEP: Pre-exposure prophylaxis for HIV
PEP: Post-exposure prophylaxis for HIV
SUD: Substance use disorder(s)
TDF: Tenofovir disoproxil fumarate
